# Effect of ecto-5′-nucleotidase (eN) in astrocytes on adenosine and inosine formation

**DOI:** 10.1007/s11302-014-9421-8

**Published:** 2014-08-17

**Authors:** Stephanie Chu, Wei Xiong, Fiona E. Parkinson

**Affiliations:** Department of Pharmacology and Therapeutics, University of Manitoba, A203-753 McDermot Avenue, Winnipeg, MB R3E 0T6 Canada

**Keywords:** Astrocyte, Neuron, Cell culture, Ecto 5′-nucleotidase, Nucleoside transport

## Abstract

ATP is a gliotransmitter released from astrocytes. Extracellularly, ATP is metabolized by a series of enzymes, including ecto-5′-nucleotidase (eN; also known as CD73) which is encoded by the gene *5NTE* and functions to form adenosine (ADO) from adenosine monophosphate (AMP). Under ischemic conditions, ADO levels in brain increase up to 100-fold. We used astrocytes cultured from *5NTE* ^+/+^ or *5NTE* ^−/−^ mice to evaluate the role of eN expressed by astrocytes in the production of ADO and inosine (INO) in response to glucose deprivation (GD) or oxygen-glucose deprivation (OGD). We also used co-cultures of these astrocytes with wild-type neurons to evaluate the role of eN expressed by astrocytes in the production of ADO and INO in response to GD, OGD, or *N*-methyl-d-aspartate (NMDA) treatment. As expected, astrocytes from *5NTE* ^+/+^ mice produced adenosine from AMP; the eN inhibitor α,β-methylene ADP (AOPCP) decreased ADO formation. In contrast, little ADO was formed by astrocytes from *5NTE* ^−/−^ mice and AOPCP had no significant effect. GD and OGD treatment of *5NTE* ^+/+^ astrocytes and *5NTE* ^+/+^ astrocyte-neuron co-cultures produced extracellular ADO levels that were inhibited by AOPCP. In contrast, these conditions did not evoke ADO production in cultures containing *5NTE* ^−/−^ astrocytes. NMDA treatment produced similar increases in ADO in both *5NTE* ^+/+^ and *5NTE* ^−/−^ astrocyte-neuron co-cultures; dipyridamole (DPR) but not AOPCP inhibited ADO production. These results indicate that eN is prominent in the formation of ADO from astrocytes but in astrocyte-neuron co-cultures, other enzymes or pathways contribute to rising ADO levels in ischemia-like conditions.

## Introduction

ATP is the energy molecule of cells. ATP levels fall during conditions characterized by reduced oxygen and glucose availability and/or increased cellular energy demand. Adenosine (ADO) is a metabolite of ATP and increased metabolism of intracellular ATP can directly increase intracellular ADO levels, which can be released from cells through equilibrative nucleoside transport proteins 1 and/or 2 (ENT1, ENT2) [1–3]. This ADO activates specific receptors of the G protein-coupled receptor family, principally A_1_ and A_2A_ receptors [4].

In addition to its role in energy requiring intracellular processes, ATP is also a signaling molecule and is released from diverse cell types, including astrocytes and neurons, in response to stimuli such as increases in cytosolic calcium levels [3, 5]. Extracellular ATP can access its receptors, broadly classified as P2X, ion channel receptors, and P2Y, G protein-coupled receptors [4, 6]. Also in the extracellular environment, ATP can be acted upon by a cascade of hydrolase enzymes to dephosphorylate ATP to ADO [7]. The final enzyme in this cascade is ecto-5′-nucleotidase (eN); the protein corresponding to this enzyme is also known as CD73 whereas the gene is known as *5NTE* [7].

ADO levels in the brain are under control of ENT1 and/or ENT2, which facilitate the movement of nucleosides, including ADO and inosine (INO), across cell membranes. Both ENT1 and ENT2 transporters can be inhibited pharmacologically by dipyridamole (DPR) or dilazep at micromolar concentrations [1]. ADO levels are also regulated by enzymes that produce or metabolize ADO, including adenosine kinase, adenosine deaminase, and 5′-nucleotidases. eN is primarily expressed in astrocytes [8] but has also been localized to synaptic membranes [9] and is an important enzyme for the extracellular formation of ADO [7].

Previous studies with cultured rat cortical astrocytes and neurons reported that, during ischemia-like conditions, astrocytes released adenine nucleotides that were metabolized extracellularly to ADO whereas neurons released ADO directly via ENT1/ENT2 [3, 10]. Using mouse hippocampal slices, neuronal overexpression of ENT1 was found to reduce extracellular ADO levels in basal and ischemia-like conditions, indicating that the extracellular pathway for ADO formation predominated in normoxic, hypoxic, and oxygen-glucose deprivation [11]. However, using hippocampal slices from *5NTE* ^−/−^ mice, a role for eN could not be demonstrated for ADO formation in response to hypoxia or oxygen-glucose deprivation [12].

The present studies were performed to examine the role of eN on astrocytes for ADO formation evoked by experimental conditions that simulate ischemia, such as glucose deprivation (GD), oxygen-glucose deprivation (OGD), and activation of *N*-methyl-d-aspartate (NMDA) glutamate receptors. We used cultures of astrocytes from *5NTE* ^+/+^ and *5NTE* ^−/−^ mice as well as co-cultures of wild type neurons with *5NTE* ^+/+^ or *5NTE* ^−/−^ astrocytes. In addition to ADO, we also measured its metabolite INO. The pharmacological tools DPR, dilazep, and α,β-methylene ADP (AOPCP), an inhibitor of eN, were used to assess the roles of ENT1/ENT2 and eN, respectively, in regulating extracellular levels of ADO and INO.

## Experimental methods

### Materials

Neurobasal media, Dulbecco’s modified Eagle medium-F12 (DMEM-F12), B-27 supplement, fetal bovine serum (FBS), l-glutamine, and antibiotic/antimycotic (penicillin, streptomycin, amphotericin B) were purchased from Invitrogen (Burlington, Ontario, Canada). [^3^H] Adenine was purchased from Perkin Elmer (Boston, MA). [^14^C] AMP was purchased from Amersham Biosciences (Baie d’Urfe, Quebec, Canada). Silica gel-coated glass plates were obtained from Fisher Scientific (Whitby, Ontario, Canada). DPR, dilazep, AOPCP, NMDA, glutamic acid, 2-deoxyglucose (2DG), and adenosine monophosphate (AMP) were purchased from Sigma-Aldrich Canada (Oakville, ON). C57Bl6 mice lacking eN expression (*5NTE* ^−/−^) mice were obtained from Dr. Linda Thompson [13] and bred locally.

### Cell culture

Primary astrocytes were cultured from cerebral cortices from *5NTE* ^+/+^ or *5NTE* ^−/−^ mice (0–3 days). Cortices were triturated several times, centrifuged at 200×*g* for 5 min, then resuspended and plated on 150-cm^2^ flasks. After 5–7 days in vitro (DIV), flasks were shaken at 300 rpm in an orbital shaker at 37 °C for 14 h to remove microglia and then plated on 12-well culture plates. Astrocytes were fed every 3 days with DMEM-F12 supplemented with 10 % FBS, 100 units/ml of penicillin, 100 μg/ml of streptomycin, and 0.25 μg/ml of amphotericin B and used at 14–21 DIV.

For primary neuron cultures, the cerebral cortices from gestational day 17 CD1 mice were isolated and triturated. Cells were incubated for 1 h at 37 °C in 150-cm^2^ flasks to allow any contaminating astrocytes to adhere. Neurons were counted and plated (30,000 per well) on top of a semi-confluent (70 %) layer of astrocytes (DIV 7–12) in 12-well plates. For 24 h prior to addition of neurons, astrocytes were pre-conditioned to Neurobasal media containing 2 % B-27 supplement, 100 units/ml of penicillin, 100 μg/ml of streptomycin, 0.25 μg/ml of amphotericin B, 500 μM l-glutamine, and 25 μM glutamic acid. After 4 days in vitro (DIV), half the media was replaced with fresh media without glutamic acid. Co-cultures were used in experiments 10 days following addition of neurons.

All procedures with animals were in accordance with animal care guidelines set by the Canadian Council on Animal Care approved by the University of Manitoba Animal Protocol Management and Review Committee.

### Ecto-5′-nucleotidase (eN) Assay

eN enzyme activity was assessed in *5NTE* ^+/+^ and *5NTE* ^−/−^ tissue samples and astrocyte cultures. To determine activity in brain tissue, cerebral cortices from *5NTE* ^+/+^ and *5NTE* ^−/−^ mice were homogenized in 0.32 M sucrose with glass/Teflon homogenizer. The homogenate was centrifuged at 1,000×*g* for 10 min and the pellet was washed twice in 0.32 M sucrose solution. The supernatants were collected at the end of each wash step, up to three times. The pooled supernatant was centrifuged at 20,000×*g* for 45 min at 4 °C. Following this, the supernatant was discarded and the pellet was resuspended in 4-2-hydroxyethyl-1-piperazineethanesulfonic acid (HEPES) buffer (110 mM NaCl, 25 mM glucose, 68.3 mM sucrose, 5.3 mM KCl, 1.8 mM CaCl_2_, 1.0 mM MgSO_4_, and 20 mM HEPES; pH 7.4) and then assayed for protein content. Samples were stored at −80 °C.

Tissue eN assay was performed with total reaction volume of 0.3 ml. This mixture consisted of 0.1 ml cortex membrane protein, prepared to final concentrations of 10 μg/ml, 0.1 ml [^14^C] AMP (300 μM), and 0.1 ml of buffer with or without AOPCP (50 μM). After 10-min incubation, samples were centrifuged for 2 min to collect supernatant to assess [^14^C] purine content by TLC and scintillation spectroscopy, as previously described [10].

For cell cultures, primary astrocytes were grown on 12-well plates. The medium was aspirated from wells and cells were gently washed twice with buffer. Cells were then incubated with 30 μM DPR in buffer for 15 min at room temperature. Following this, 1.85 kBq [^14^C] AMP (10 μM) containing 30 μM DPR with or without 50 μM AOPCP was added to cells for 10 min at room temperature. DPR was included in the assays to minimize cellular uptake of any [^14^C] ADO formed. After incubation, the extracellular medium was extracted and assayed for [^14^C] purines by TLC. Cells were lysed with 1.0 M NaOH and measured for intracellular [^14^C] purines and protein content.

### Nucleoside release assays

All experiments with astrocytes or co-cultures were performed with physiological buffer that contained a final concentration of 25 mM HEPES, 2.9 mM KCl, 1.2 mM MgCl_2_, 4.9 mM KCl, 1.4 mM KH_2_PO_4_, 1 mM CaCl_2_, 118 mM NaCl, and 11 mM glucose, at pH 7.4, and an osmolarity of 300 ± 10 mOsm. Cells were washed twice with buffer (37 °C) and then incubated with 13.7 kBq [^3^H] adenine for 30 min at 37 °C. The [^3^H] adenine is taken up by cells and is metabolized to [^3^H] adenine nucleotides [10]. To assay nucleoside release in response to ischemia-like conditions, cells were washed to remove extracellular [^3^H] adenine then treated with buffer (control), glucose deprivation (GD), or oxygen-glucose deprivation (OGD) in the absence or presence of AOPCP (50 μM), to inhibit eN, or DPR (30 μM), to inhibit ENT1 and ENT2. In some experiments, dilazep (100 μM) was used in place of DPR. For GD, cells were treated for 30 min at 37 °C with buffer in which glucose was replaced with 2DG (10 mM) to inhibit glycolysis. For OGD treatment, cells were treated with 2DG containing buffer and were placed in a humidified chamber containing 95 % N_2_ and 5 % CO_2_ for 1 h at 37 °C. Oxygen content in the chamber was monitored with a ProOx 110 controller and maintained at 2 %.

To assay nucleoside release in response to glutamate receptor activation, cells were washed to remove extracellular [^3^H] adenine then treated with buffer (control), NMDA (100 μM), DPR (30 μM), or AOPCP (50 μM), separately or in combination, for 30 min at 37 °C. After incubation, the supernatants were collected and 0.4 ml was quantified for extracellular [^3^H] purines by TLC and scintillation spectrometry. Cells were dissolved in 0.35 ml 1 M NaOH overnight at 37 °C; 0.2 ml of the cell lysate was counted for intracellular radioactivity and 0.1 ml for protein content.

### Statistical analysis

All data are reported as means ± SEM. Data were obtained from a minimum of three independent culture preparations; reported *n* values are the total number of replicates. Statistical significance between two means was determined by unpaired Student’s *t* test; statistical significance between three or more means was determined by one-way ANOVA with Tukey’s post hoc tests.

## Results

### AOPCP inhibits ADO production from AMP in cortical membranes and cortical astrocytes from 5NTE^+/+^ but not 5NTE^−/−^ mice

**Fig. 1:**
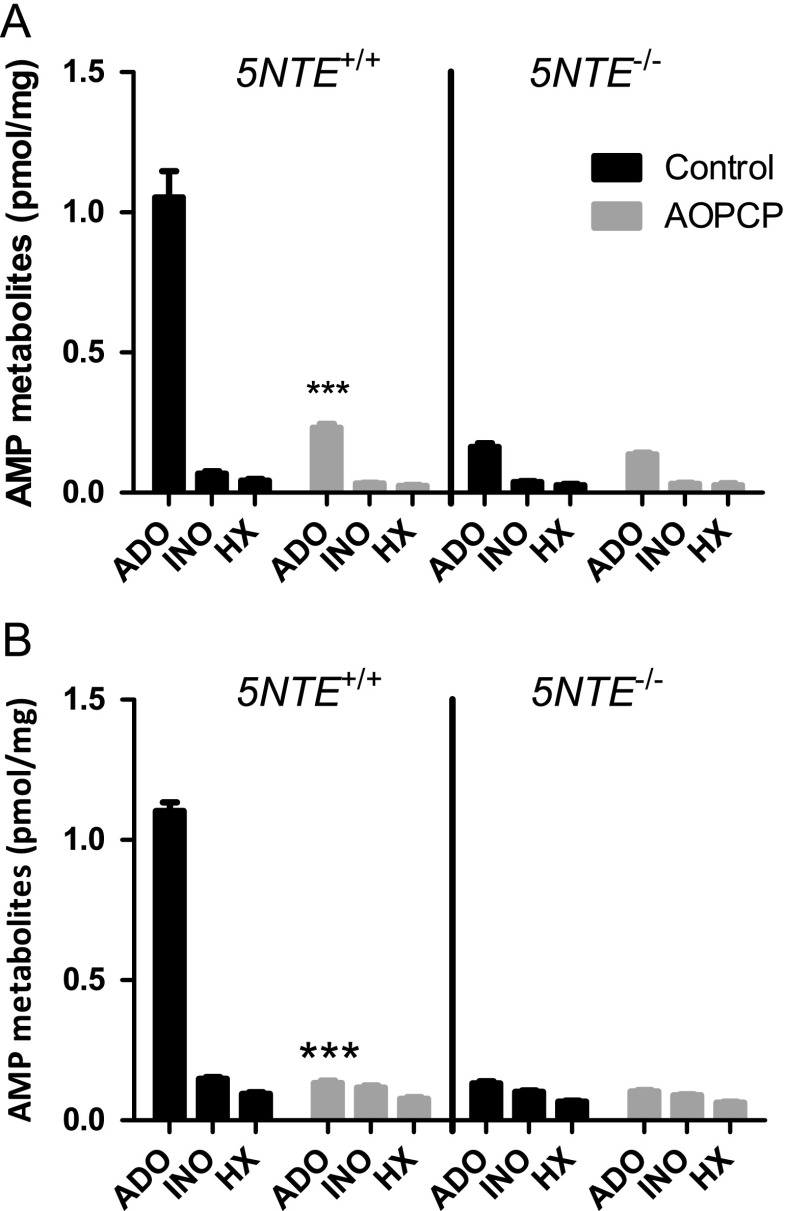
Ecto-5′ nucleotidase (eN) assays in cortical tissues and cultured astrocytes from *5NTE* ^+/+^ or *5NTE* ^−/−^ mice. **a** Membrane proteins were extracted from tissue and incubated with 10 μM [^14^C] AMP, with or without 50 μM AOPCP for 10 min. Supernatants were collected and ADO, INO, and HX were separated by TLC then quantified by scintillation spectroscopy. Data are expressed as means ± SEM (*n* = 6). A significant effect of AOPCP to inhibit ADO production in Wt tissue was identified by two-way ANOVA, followed by Bonferroni test for multiple comparisons. ***P < 0.001. **b** Cultured astrocytes were first treated with 30 μM DPR for 15 min and then treated for 10 min with a reaction mixture containing final concentrations of 10 μM [^14^C] AMP and 30 μM DPR with or without 50 μM AOPCP. Afterwards, supernatant was collected for TLC and quantified by scintillation spectrometry. Data are expressed as mean ± SEM (*n* = 18). A significant effect of AOPCP to inhibit ADO production in *5NTE* ^+/+^ cells was identified by two-way ANOVA, followed by Bonferroni test for multiple comparisons. ***P < 0.001

For tissue eN assays, membrane proteins from *5NTE* ^+/+^ and *5NTE* ^−/−^ cerebral cortex was incubated with AMP. As shown in Fig. 1a, ADO was the most abundant purine formed and 6-fold more ADO was formed with *5NTE* ^+/+^ tissue than with *5NTE* ^−/−^ tissue. AOPCP significantly inhibited the formation of ADO in *5NTE* ^+/+^ tissue, but not in *5NTE* ^−/−^ tissue. In the presence of AOPCP, ADO formation was not significantly different between *5NTE* ^+/+^ and *5NTE* ^−/−^ samples.

The activity of membrane-bound eN was assayed in cultures of *5NTE* ^+/+^ and *5NTE* ^−/−^ astrocytes (Fig. 1b). Similar to the results with cortical membranes, *5NTE* ^+/+^ astrocytes produced 8-fold more ADO from AMP than *5NTE* ^−/−^ astrocytes. While AOPCP significantly inhibited ADO formation by *5NTE* ^+/+^ astrocytes, it had no significant effect on the low levels of ADO formed by *5NTE* ^−/−^ astrocytes.

### In ischemia-like conditions, 5NTE^+/+^ astrocytes produce more ADO but similar INO compared to 5NTE^−/−^ astrocytes

**Fig. 2:**
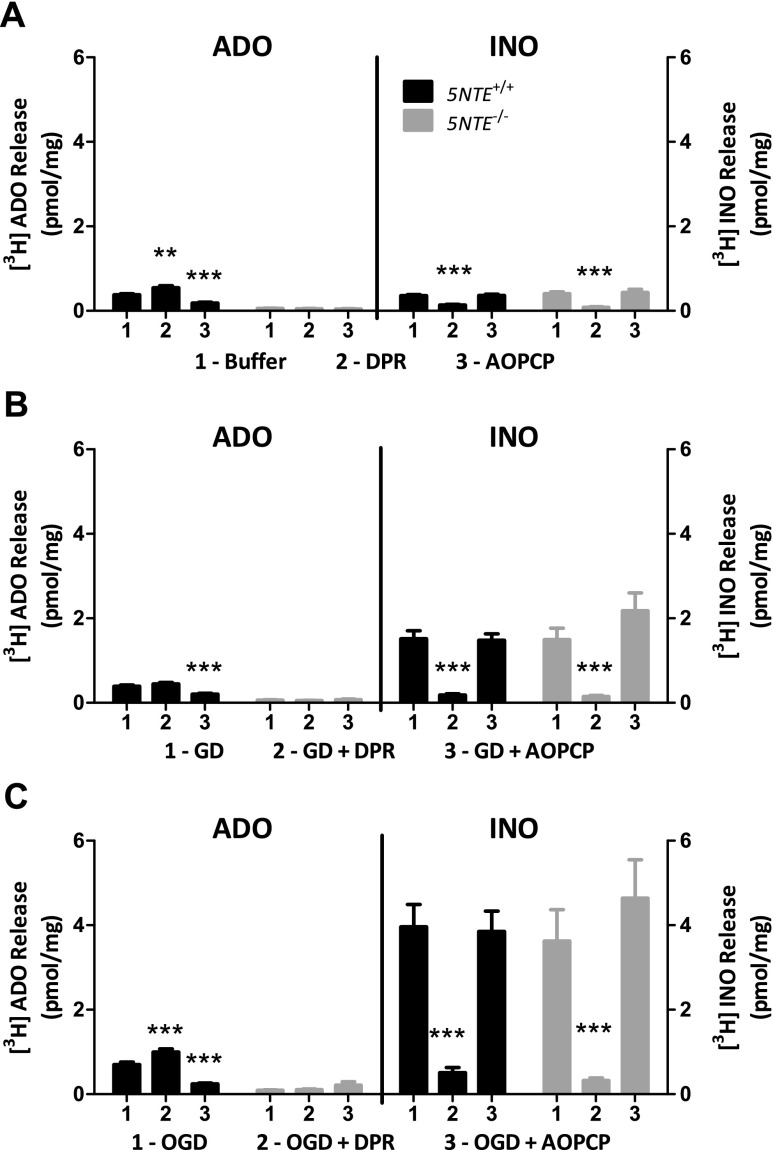
ADO and INO release from *5NTE* ^+/+^ or *5NTE* ^−/−^ astrocyte cultures in energy-depleting conditions. Cells were pre-incubated with [^3^H] adenine and then treated with **a** buffer, **b** glucose deprivation (*GD*), or **c** oxygen-glucose deprivation (*OGD*) in the absence or presence of 30 μM DPR or 50 μM AOPCP. For buffer and GD conditions, supernatants were collected after 30 min. For OGD conditions, supernatants were collected after 1 h in a humidified chamber containing 95 % N_2_ and 5 % CO_2_ at 37 °C. [^3^H] Purines were separated by TLC and analyzed by scintillation spectroscopy. Analysis was done by one-way ANOVA with post hoc analysis using Dunnett’s multiple comparison test. Data are expressed as means ± SEM (*n* = 18–20). ***P < 0.001; **P < 0.01, *P < 0.05 compared with buffer, GD, or OGD treatments

Primary cultured astrocytes from *5NTE* ^+/+^ and *5NTE* ^−/−^ mice were compared to assess ADO and INO release under physiological buffer (control), GD, or OGD conditions. Compared to *5NTE* ^+/+^ astrocytes, the *5NTE* ^−/−^ cells produced significantly less ADO in all three conditions (Fig. 2a–c). In contrast, there were no significant differences in INO release between *5NTE* ^+/+^ and *5NTE* ^−/−^ cells in control, GD, or OGD conditions (Fig. 2a–c). In *5NTE* ^+/+^ cells, GD and OGD produced greater increases in INO than ADO levels (Fig. 2b, c).

DPR, which inhibits equilibrative nucleoside transporters 1 and 2, produced a significant increase in ADO in buffer-treated and OGD-treated *5NTE* ^+/+^ astrocytes (Fig. 2a, c). No effect of DPR on ADO levels was observed in *5NTE* ^−/−^ astrocytes (Fig. 2a–c). In contrast, DPR reduced INO levels in both *5NTE* ^+/+^ and *5NTE* ^−/−^ astrocytes and this inhibition was evident in buffer-, GD-, and OGD-treatment conditions (Fig. 2a–c). Similar to DPR, dilazep also inhibited INO levels in *5NTE* ^+/+^ astrocytes (data not shown).

AOPCP was observed to inhibit ADO production in *5NTE* ^+/+^ astrocytes in buffer-, GD-, and OGD-treatment conditions (Fig. 2a–c). In contrast, AOPCP had no effect on ADO production in *5NTE* ^−/−^ astrocytes and also had no effect on INO levels in either cell type (Fig. 2a–c).

### In ischemia-like conditions, co-cultures of wild type neurons with 5NTE^+/+^ astrocytes produced more ADO but similar INO compared to co-cultures of wild type neurons with 5NTE^−/−^ astrocytes

**Fig. 3:**
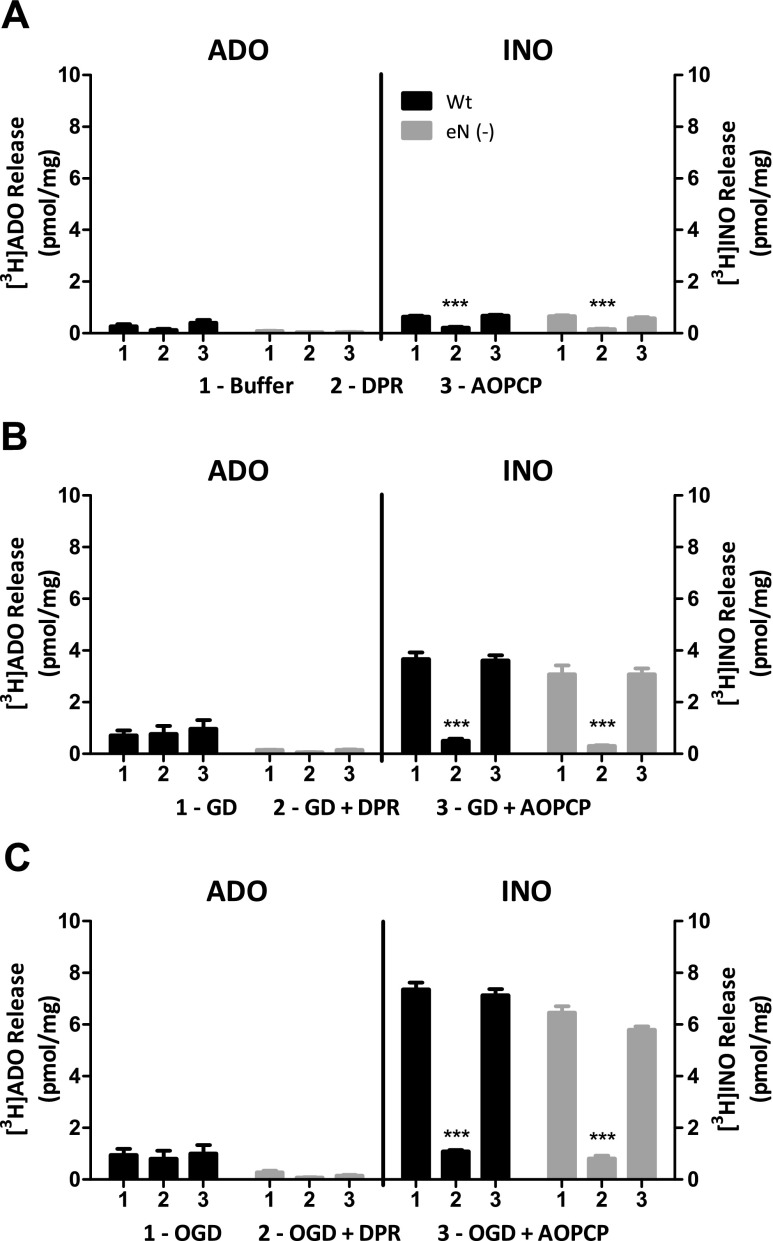
ADO and INO release from CD1 neuron-*5NTE* ^+/+^ astrocyte (Wt) and CD1 neuron-*5NTE* ^−/−^ astrocyte (eN (−)) co-cultures. Co-cultures were incubated with [^3^H] adenine then treated with **a** buffer, **b** glucose deprivation (*GD*), or **c** oxygen-glucose deprivation (*OGD*) in the absence or presence of 30 μM DPR or 50 μM AOPCP. For buffer and GD conditions, extracellular fluid was collected after 30 min. For OGD conditions, the supernatants were collected after 1 h in a humidified chamber containing 95 % N_2_ and 5 % CO_2_ at 37 °C. [^3^H] Purines were separated by TLC and analyzed by scintillation spectroscopy. Data are expressed as means ± SEM (*n* = 20–24). Statistical analysis between control and treatment groups for CD1-*5NTE* ^+/+^ or CD1-*5NTE* ^−/−^ co-cultures was performed by one-way ANOVA and post hoc analysis with Tukey’s tests. ***P < 0.001 compared to CD1-*5NTE* ^+/+^ or CD1-*5NTE* ^−/−^ co-culture buffer, GD, or OGD treatments

Neuron-astrocyte co-cultures consisting of wild-type neurons cultured with astrocytes from either *5NTE* ^+/+^ or *5NTE* ^−/−^ mice were compared to assess ADO and INO release under physiological buffer (control), GD, or OGD conditions. Under these treatment conditions, co-cultures with eN-competent cells produced more ADO than co-cultures deficient in eN (Fig. 3a–c). In contrast, for each treatment condition, INO production was similar between cell cultures (Fig. 3a–c). Using co-cultures, ADO formation was not significantly affected by DPR or AOPCP (Fig. 3a–c). In contrast, INO production was consistently reduced by DPR, but not AOPCP (Fig. 3a–c).

### NMDA-evoked release of ADO and INO was reduced in astrocyte-neuron co-cultures containing 5NTE^−/−^ astrocytes relative to co-cultures containing 5NTE^+/+^astrocytes

**Fig. 4:**
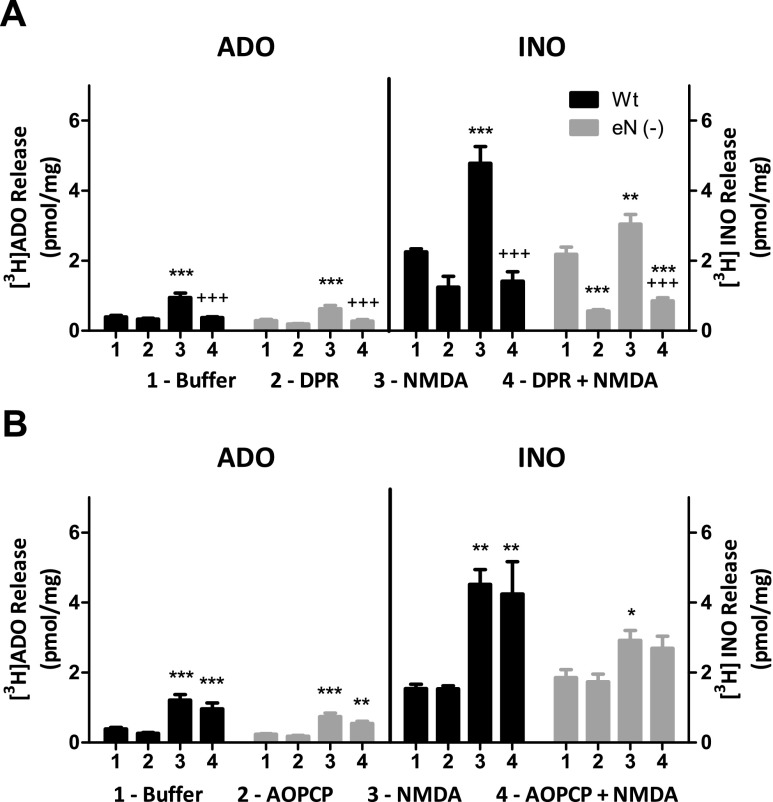
ADO and INO release from CD1 neuron-*5NTE* ^+/+^ astrocyte (Wt) and CD1 neuron-*5NTE* ^−/−^ astrocyte (eN (−)) co-cultures. Co-cultures were incubated with [^3^H] adenine then treated with **a** buffer, 30 μM DPR, 100 μM NMDA, or DPR + NMDA or **b** buffer, 50 μM AOPCP, 100 μM NMDA, or AOPCP + NMDA. Supernatants were collected after 30-min incubation at 37 °C. [^3^H] Purines were separated by TLC and analyzed by scintillation spectroscopy. Data are expressed as means ± SEM (*n* ≥ 9). Statistical analysis was performed by one-way ANOVA and post hoc analysis with Tukey’s tests. ***P < 0.001; **P < 0.01, *P < 0.05 compared with buffer-treated CD1-*5NTE* ^+/+^ or CD1-*5NTE* ^−/−^ co-cultures. ^+++^ *P* < 0.001 compared to NMDA-treated CD1-*5NTE* ^+/+^ or CD1-*5NTE* ^−/−^ deficient co-cultures

Neuron-astrocyte co-cultures consisting of wild-type neurons cultured with astrocytes from either *5NTE* ^+/+^ or *5NTE* ^−/−^ mice were treated with buffer or NMDA and evaluated for ADO and INO release. NMDA treatment increased both ADO and INO production in both wild-type and eN-deficient co-cultures (Fig. 4a, b). DPR significantly reduced NMDA-evoked ADO and INO release from both wild-type and eN-deficient co-cultures (Fig. 4a, b). In contrast, AOPCP had no significant effect on either ADO or INO in either co-culture (Fig. 4a, b).

## Discussion

The main findings of this study were that astrocytes cultured from *5NTE* ^−/−^ mice produced virtually no ADO in the presence of buffer or in response to GD or OGD treatment, in contrast to *5NTE* ^+/+^ astrocytes. A similar lack of ADO production was observed in co-cultures of wild-type neurons with *5NTE* ^−/−^ astrocytes treated with these ischemia-like conditions. However, NMDA treatment did evoke ADO production in co-cultures of wild-type neurons with *5NTE* ^−/−^ astrocytes. Regarding INO production from astrocyte cultures or astrocyte-neuron co-cultures, the deficiency of eN did not have an effect.

ADO formation from AMP in normoxic and normoglycemic cortical membrane preparations and cortical astrocyte cultures was largely dependent upon eN. This was confirmed by the use of AOPCP to inhibit eN activity in *5NTE* ^+/+^ preparations and by the substantially reduced quantities of ADO produced by *5NTE* ^−/−^ preparations. The residual ADO present in *5NTE* ^−/−^ membranes and astrocyte cultures (Fig. 1) may indicate a small contribution of alternate enzyme activities, such as tissue non-specific alkaline phosphatase, as reported previously [12]. The eN assay conditions were relatively clear of contamination from enzymes producing INO or hypoxanthine. The small quantities of these purines produced in astrocyte cultures (Fig. 1b) may indicate that the concentration of DPR used (30 μM) was not completely effective in preventing cellular uptake of extracellular ADO or release of intracellularly formed purines.

Previously, we reported that the greatest amount of ADO evoked from astrocytes occurred with inhibition of both glycolysis and oxidative phosphorylation for 60 min [3]. In the present study, we inhibited glycolysis by exposing astrocyte cultures to 2DG, a competitive inhibitor of hexokinase, a key enzyme in glycolysis. We inhibited oxidative phosphorylation by placing astrocytes in an oxygen-depleted humidified chamber. eN activity was important for ADO production in basal, GD, and OGD conditions. First, extracellular ADO was low, almost undetectable, in cultures of *5NTE* ^−/−^ astrocytes. Second, AOPCP decreased ADO levels in *5NTE* ^+/+^ astrocytes. Third, DPR increased ADO indicating that ADO was formed extracellularly and DPR-sensitive nucleoside transporters mediated cellular uptake and removal of ADO. Because AOPCP significantly reduced ADO levels in *5NTE* ^+/+^ cells but extracellular ADO levels in *5NTE* ^−/−^ cells were insensitive to GD and OGD treatments, it appears that eN was an important source of extracellular ADO even under ischemia-like conditions that inhibit intracellular ATP production.

Astrocyte cultures produced extracellular INO in addition to ADO. Consistently, in both *5NTE* ^+/+^ and *5NTE* ^−/−^ astrocyte cultures, INO levels were reduced by DPR but not by AOPCP, indicating that INO is produced intracellularly and released via nucleoside transporters under basal and ischemia-like conditions. Dilazep, an ENT1 and ENT2 inhibitor structurally unrelated to DPR, also inhibited extracellular INO levels in *5NTE* ^+/+^ astrocyte cultures. As ADO levels were increased, but INO levels were decreased by DPR, we conclude that INO was produced by an intracellular pathway in contrast to the extracellular pathway for ADO formation. Further, both the presence of AOPCP and the absence of eN inhibited ADO but had no effect on INO. Thus, INO was not produced simply as a downstream metabolite of ADO in astrocyte cultures.

Using astrocyte-neuron co-cultures, more ADO was produced in cultures containing *5NTE* ^+/+^ astrocytes than in cultures containing *5NTE* ^−/−^ astrocytes. ADO production was virtually undetectable in co-cultures that contained *5NTE* ^−/−^ astrocytes. In contrast, INO production was similar between the two co-cultures. As in astrocyte cultures, INO was significantly reduced by DPR, indicating an intracellular source of this purine. In contrast to *5NTE* ^+/+^ astrocyte cultures, ADO was not significantly affected by AOPCP or DPR in *5NTE* ^+/+^ astrocyte-neuron co-cultures in control or ischemia-like conditions. Thus, neurons appear to contribute additional pathways for ADO production and removal that counteract the contributions of astrocytic eN and DPR-sensitive nucleoside transporters.

In previous studies, we treated hippocampal slices with hypoxia or OGD and found that ADO A_1_ receptor activity, a measure of extracellular ADO levels, was reduced by overexpression of ENT1, a DPR-sensitive nucleoside transporter, indicating an extracellular site of ADO production [11]. However, the hypoxia or OGD-induced increases in ADO A_1_ receptor activity were not affected by the absence of eN, indicating either another extracellular enzyme, such as tissue non-specific alkaline phosphatase, or another release mechanism for ADO [12]. The present study also indicates a role for additional sources of ADO in astrocyte-neuron co-cultures treated with GD or OGD.

In experiments using biosensors to detect ATP and ADO, OGD was found to increase extracellular levels of both of these purines in hippocampal slices [14]. While nucleoside transport inhibitors increased ADO levels, indicating extracellular formation of ADO, the ATP and ADO increases appeared to result from independent mechanisms; thus, it was concluded that ADO was not simply a metabolite of ATP release [14]. Although the role of eN was not addressed directly, as ADO production was not correlated to ATP levels, it may have also been independent of eN activity. With respect to INO formation, biosensors were used to compare ADO and INO formation in hippocampal slices [15]. Hypoxia increased both nucleosides but had a greater effect on ADO than INO, in contrast to the cell culture data reported here.

Fast-scan cyclic voltammetry has been used to measure adenosine levels in brain slices and in vivo brain cortex [16, 17]. Mechanical or brief electrical stimulations increased ADO levels; partial reductions in ADO levels were achieved with ARL 65156 and AOPCP or POM-1, drugs that inhibit extracellular metabolism of adenine nucleotides. Using these stimulation conditions, there was no evidence for release of cytoplasmic ADO as nucleoside transport inhibitors had no effect.

In contrast to GD and OGD, exposure of astrocyte-neuron co-cultures to NMDA, an agonist for the NMDA subtype of glutamate receptors, stimulated significant increases in extracellular levels of both ADO and INO in co-cultures containing either *5NTE* ^+/+^ or *5NTE* ^−/−^ astrocytes. For both ADO and INO, DPR, but not AOPCP, significantly reduced NMDA-induced increases. These data indicate that NMDA treatment produces both ADO and INO that are formed intracellularly and released via nucleoside transporters. Previous studies have shown that NMDA has little effect on ADO production in astrocyte cultures, despite reports that astrocyte cultures express functional NMDA receptors [18, 19]. Therefore, our results indicate that NMDA selectively activates neurons and stimulates cytosolic formation of ADO and INO, which are subsequently effluxed via membrane transporters.

A previous study, which used rat cortical astrocytes and rat cortical neurons in co-culture, reported that NMDA significantly increased INO but did not significantly increase ADO production [20]. In these rat co-cultures, AOPCP, but not DPR, inhibited ADO production. The difference between these earlier results and the present findings could be species of origin of the cells, or the number of astrocytes used to initiate the co-cultures. In the earlier study, astrocytes were confluent prior to the addition of neurons [20] whereas in the present study, astrocytes were sub-confluent, and this may have reduced the contribution of eN to ADO formation and reduced the inhibitory effect of AOPCP in the present study.

Brain ischemia arises from brain trauma, stroke, or cardiac arrest. Extensive research has focused on neurons as these cells are particularly sensitive to ischemic insult [21]. Astrocytes are less sensitive to ischemic damage than neurons [21, 22], in part, because as non-excitable cells, astrocytes have a reduced rate of ATP consumption than neurons. Further, these cells contain glycogen, which allows ATP synthesis to continue to a limited extent during ischemia. Previously, we reported the enhanced rate of ATP depletion in neurons compared to astrocytes in ischemia-like conditions [3]. These differences in rates of ATP depletion may, in part, explain the intracellular formation of ADO by neurons but not by astrocytes.

In summary, this study shows that eN activity by astrocytes produces ADO under basal conditions and in response to GD and OGD. It has a reduced role in ADO formation in astrocyte-neuron co-cultures and does not contribute to ADO produced in response to NMDA treatment of astrocyte-neuron co-cultures.
